# Exploring the Mechanisms of Ecological Land Change Based on the Spatial Autoregressive Model: A Case Study of the Poyang Lake Eco-Economic Zone, China 

**DOI:** 10.3390/ijerph110100583

**Published:** 2013-12-31

**Authors:** Hualin Xie, Zhifei Liu, Peng Wang, Guiying Liu, Fucai Lu

**Affiliations:** 1 Institute of Poyang Lake Eco-economics, Jiangxi University of Finance and Economics, Nanchang 330013, China; E-Mails: liuzhifei1972@126.com (Z.L.); landuse2008@126.com (P.W.); liuguiying_2013@126.com (G.L.); fucai@126.com (F.L.); 2 School of Economics and Management, Jiangxi Agriculture University, Nanchang 330045, China

**Keywords:** ecological land, public health and safety, land use change, ecological management, spatial autocorrelation, spatial autoregressive model

## Abstract

Ecological land is one of the key resources and conditions for the survival of humans because it can provide ecosystem services and is particularly important to public health and safety. It is extremely valuable for effective ecological management to explore the evolution mechanisms of ecological land. Based on spatial statistical analyses, we explored the spatial disparities and primary potential drivers of ecological land change in the Poyang Lake Eco-economic Zone of China. The results demonstrated that the global Moran’s I value is 0.1646 during the 1990 to 2005 time period and indicated significant positive spatial correlation (*p* < 0.05). The results also imply that the clustering trend of ecological land changes weakened in the study area. Some potential driving forces were identified by applying the spatial autoregressive model in this study. The results demonstrated that the higher economic development level and industrialization rate were the main drivers for the faster change of ecological land in the study area. This study also tested the superiority of the spatial autoregressive model to study the mechanisms of ecological land change by comparing it with the traditional linear regressive model.

## 1. Introduction

Land use is defined as the activities of human beings that use their labor to obtain material goods and services. Land use activities are generally manifested as processes of exchange and conversion between humans and substances, energy, and information [1,2]. As the core project of the International Human Dimensions Program (IHDP) and the International Geosphere-Biosphere Program (IGBP), the Global Land Project (GLP) has been gaining attention from scholars because of its close relationship with social, economic, and environmental issues [3,4,5,6,7,8,9]. Land Use and Land Cover Change (LUCC) is one of the important research topics of the GLP. LUCC not only brings about tremendous changes in the landscape structure, but also affects the nutrient cycling and energy flow and has a profound impact on the regional biosafety and ecological balance. In contrast with construction land meeting the need of urban development and farmland supporting food security, ecological land is defined as the land resources that provide natural ecosystem services and maintain regional ecological security. It is one of the basic resources and conditions for the survival of humans and other organisms. Ecological land plays an irreplaceable role in the healthy maintenance of urban or regional ecosystems. Furthermore, ecological land makes the lives of urban residents better by providing a variety of ecosystem services, including the provision of clean drinking water, regulating climate, and controlling floods. In the 21st century, urbanization is an irrepressible trend as human society develops. Urban expansion cannot be avoided; consequently, many ecological lands, which play a pivotal role in ecological service functions, will be converted to construction land [10]. Urban expansion has caused extremely large impacts on regional and global ecosystems [11]. Protecting ecological land, which is gradually improving, and restoring the serious ecological damage zone and refunding the natural land is significant because it can maintain the ecological balance, improve the quality of a regional eco-environment, promote the harmonious co-existence of man and nature, and realize sustainable economic and social growth.

In China, the land use management objectives are to ensure survival, economic development, and environmental protection. The object of environmental protection in China now still rests on the traditionally rational level, and is less considered in land use planning practices. Because of the impacts of policies that can be characterized as taking into account food problems first, and economical construction and ecological protection last, land administration departments in China have long highlighted the functions of carrying, producing, and utilizing land resources [12]. The land management department in China has not paid sufficient attention to land functions that support and maintain the stability of natural and artificial ecosystems. Because the current land classification system in China has no classification for ecological land, the goals of environmental protection cannot be reflected in the various levels of land-use planning programs. Because of the increasing demand for construction land, ecological land in China faces the threat of agricultural exploitation because of the policy “cultivated land requisition-compensation balance”.

The Poyang Lake Region of China is an important eco-zone that was delineated by the World Wide Fund for Nature (WWF). Since the beginning of the 1990s, however, rapid industrial development coupled with multiple pressures—including population growth, energy consumption, and environmental degradation—has caused increasingly prominent ecological and environmental problems in the Poyang Lake Eco-economic Zone. It is necessary to study the evolved mechanisms of ecological land in the Poyang Lake Eco-economic Zone because the information is highly valuable for understanding ecological land and the vast eco-environment effects of the LUCC. It is particularly beneficial for enriching the spatial strategy of maintaining regional ecological security. It is urgent to study the evolutionary characteristics and the factors that influence ecological land change in the Poyang Lake Eco-economic Zone.

Most of the traditional methods that measure the spatial difference in land-use changes assume that the studied spatial entities are independent and include little consideration of spatial correlations. It is difficult to reflect the overall regional differences and spatial heterogeneity of land-use changes. Spatial statistical methods, including exploratory spatial data analysis (ESDA) and spatial autoregressive analysis, can address spatial autocorrelation problems. Among them, ESDA is one of a series of spatial data analysis methods and technologies [13]. Through the description and visualization of the spatial pattern of land use change, ESDA can determine the spatial concentration and heterogeneity and reveal the spatial interaction mechanisms between research objects. To date, the ESDA method has been widely used in regional economic development, fire risk analyses, and studies of house price patterns, water environment management, land-use change studies, and other research areas [13,14,15,16,17,18,19]. When determining the driving forces of land use change by a classic linear regression model, we usually ignore the influence of spatial autocorrelation. The spatial autoregressive model is suitable for processing spatial data and providing a statistically reasonable method. Thus, the advantage is that the spatial autoregressive model identifies the spatial variation in the driving forces. The spatial autoregressive model is also widely applied for studying point-source pollution, groundwater nitrate concentration, ecology, water quality, and land use change [19,20,21,22,23,24,25,26,27].

The main purposes of this study are: (1) to explore the spatial correlation and heterogeneity of ecological land change in the Poyang Lake Eco-economic Zone, (2) to find the potential influencing factors of ecological land change in the study area, and (3) to test the superiority of the spatial autoregressive model by comparing it with the traditional linear regressive model.

## 2. Materials and Methods

### 2.1. Study Area

**Figure 1:**
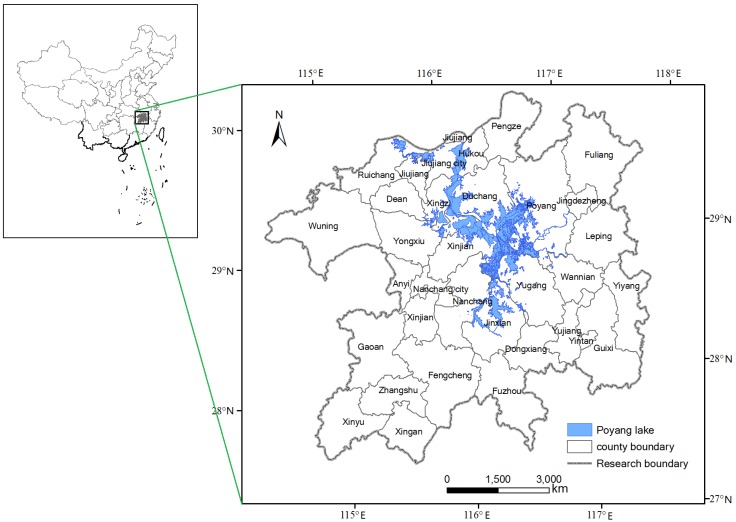
Map of the location of the Poyang Lake Eco-economic Zone in China.

The Poyang Lake Eco-economic Zone (28°30′N–30°06′N, 114°29′E–117°25′E) is located in the northern part of Jiangxi Province, China and is approximately 51,200 km^2^ in area (Figure 1). In natural conditions, the study area belongs to a subtropical humid climate zone with an annual average temperature of 16 to 18 °C and rainfall average of 1,600 mm. The main soil types include humid-thermo ferralitics, red earths, and yellow earths. The Poyang Lake Eco-economic Zone study area includes 38 counties and had a population of 20.53 million and a GDP of 5,544.76 billion Yuan (RMB) in 2010. The study area provides ecosystem services of flood-water storage, climate regulation, and pollution degradation. Unreasonable land-use patterns have led to heavier soil erosion, loss of biodiversity, deforestation, and degradation of wetland functions. The Poyang Lake Eco-economic Zone is in a stage of rapid urbanization and industrialization. This is not only a golden period of economic development but also a critical period of increasing resources and energy pressures, which deteriorates ecosystems.

### 2.2. Data

The land-use data used in this study were derived from the 1:100,000 national land-use database of the Data Center for Resources and Environmental Sciences of the Chinese Academy of Sciences (RESDC), which has successively collected data every five years since the late 1980s (henceforth referred to as 1990) [28]. To verify the accuracy of ecological land data, we used 1,000 samples for verification by random spatial sampling. The results of the validation study demonstrate that the land-use data for 1990, 2000, and 2005 have an accuracy of greater than 90%. The classification accuracy of land-use data for 1995, however, is not high at only 69.12%. Thus, the land-use data for 1990, 2000, and 2005 were selected to study the mechanisms of ecological land changes. The ecological land types were divided into forestland, grassland, and wetland. DEM data with a spatial resolution of 30 m × 30 m were also collected from the Data Center for Resources and Environmental Sciences of the Chinese Academy of Sciences. All of the data were resampled to a resolution of 100 m × 100 m and aggregated at the county level to match the counties. Socioeconomic data at the county level was derived from the statistics yearbook in the Jiangxi Province from 2000 to 2010 and included the total population, rural population, total employees, employees in the primary sector, net *per capita* income of farmers, number of rural laborers, and GDP.

### 2.3. Methods

#### 2.3.1. Global Spatial Autocorrelation

There are two different scales to measure regional spatial dependence: The global spatial autocorrelation and the local spatial autocorrelation. The global spatial autocorrelation is a measure of the overall clustering of some geographical phenomena. Three common statistical algorithms in the global spatial autocorrelation are Moran’s I, Geary’s C, and Getis’s G [2,3,4,5,6,7,8,9,10,11,12,13,14,15,16,17,18,19,20,21,22,23,24,25,26,27,28,29,30,31,32]. Moran’s I is a weighted correlation coefficient that is used to detect departures from spatial randomness. Moran’s I is used to determine whether neighboring areas are more similar than would be expected under the null hypothesis. In this paper, Moran’s I was used to measure the global spatial autocorrelation of ecological land change in the Poyang Lake Eco-economic Zone of China. Moran’s I is defined as:

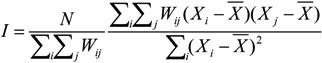
(1)
where N is the number of spatial units indexed by i and j, X is the ratio of ecological land change, *X* is the mean of X, and *W_ij_* is the matrix of weights that, in some cases, is equivalent to a binary matrix with ones in position i,j whenever observation i is a neighbor of observation j and zero otherwise.

Negative (positive) values of Moran’s I indicate a negative (positive) spatial autocorrelation. Moran’s I values range from −1 (indicating perfect dispersion) to +1 (perfect correlation). A zero value indicates a random spatial pattern. For statistical hypothesis testing, Moran's I values can be transformed to a Z_score_, in which values greater than 1.96 or less than −1.96 indicate a spatial autocorrelation that is statistically significant at the 5% level. The significance of Moran’s I can be judged by calculating the variance of Moran’s I and then comparing the following statistic to the standard normal distribution. The Zscore is defined as:


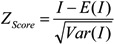
(2)

In Equation (2), E (I) is the expected value of Moran’s I and Var (I) is the variance of Moran’s I.

#### 2.3.2. Local Spatial Autocorrelation

The local indicator of spatial association (LISA) to evaluate the clustering in the individual units is calculated using the local value Moran’s I for each spatial unit and evaluating the statistical significance for each I_i_. The LISA is defined as:

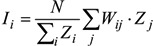
(3)
I_i_ is the indicator of spatial association (LISA), Z is the deviation of the variable of the ratio of ecological land change with respect to the mean, and N is the number of analysis units in the map.

A scatter plot of Moran’s I in a spatial autocorrelation analysis can directly reflect the degree of spatial autocorrelation [30]. The first (high-high) and third (low-low) quadrant in the Moran’s I scatter plot represent spatial clustering of similar values (either high or low), which is expressed as a positive local spatial autocorrelation. The second (low-high) and fourth (high-low) quadrant in the Moran’s I scatter plot represent clustering of dissimilar values (e.g., a location with high values surrounded by neighbors with low values), which is expressed as a negative local spatial autocorrelation. Local spatial clusters, which are sometimes referred to as hot spots, may be identified as those locations or sets of contiguous locations for which the LISA is significant. The LISA definition presented here is easy to implement and readily lends itself to visualization [30]. In this study, we use the ecological land change ratio as the dependent variable, and two periods from 1990 to 2005 are considered at the Poyang Lake Economic Zone county level. The equation for ecological land change ratio is as follows:

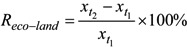
(4)
where t_1_, t_2_ are, respectively, the early year and last year (t_2_ > t_1_), and *x*_*t*_1__, *x*_*t*_2__ represent the ecological land area at time t_1_ and t_2_, respectively. 

#### 2.3.3. Spatial Autoregressive Model

The most general formulation of a spatial autoregressive model is Euqation (5) [25,29,30,31]:

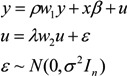
(5)
where *y* is an (*n* × 1) vector that represents the dependent variable, x is an (*n* × *k*) matrix that represents the *k* − 1 independent variables, β is a (*k* × *1*) vector of error terms that are presumed to have a covariance structure, ρ is a spatial lag parameter to be estimated, *W* is an (*n* × *n*) “weights” matrix that defines the “neighborhood” structure in the spatial process such that *Wu* is an *n* × 1 vector of spatial lags of the model disturbance term *u*, and ɛ is an (*n* × 1) vector of independently (but not necessarily identically) distributed errors. From the general formulation of the spatial autoregressive model, we can derive four spatial regressive models. When ρ = 0 and λ = 0, Euqation (5) transforms into the ordinary linear regression model, which implies that there is no spatial attribute influence in the regression model.

When ρ ≠ 0, β = 0, and λ = 0, Euqation (5) transforms into the first-order spatial autoregressive model, which is similar to the first-order autoregressive model in the time series analysis. It reflects the related characteristics of variables in space, which implies that the dependent variable in the research area is impacted by the dependent variable in its adjacent areas.

When ρ ≠ 0, β ≠ 0, and λ = 0, Euqation (5) transforms into the mixed first-order spatial autoregressive model, namely the spatial lag model (SLM). In this model, the dependent variable (*Y*) in the research area is not only related with independent variables (X) in its area but also with the dependent variable (WY) in its adjacent areas. The SLM probes into the variables as if there is a diffusion phenomenon of each variable in a region (overflow effect).

When ρ = 0, *β* ≠ 0, and λ ≠ 0, Equation (5) transforms into the residual spatial autoregressive model, namely the spatial error model (SEM). In this model, the dependent variable (Y) in the research area is not only related with the independent variables (X) in its area but also with the independent variable (WY) and the dependent variables (WX) in adjacent areas.

The traditional R^2^ measure of fit is not suitable for the spatial regressive model. Instead, a number of so-called pseudo R^2^ measures can be computed. In spatial statistics, the pseudo R^2^ is defined as the ratio of the variance of the predicted values to the variance of the observed values for the dependent variable [25,30]. This variance ratio is equivalent to the R^2^ in the standard regression model but not in the spatial lag model and the SEM [25,30]. Measure indicators of fit for the spatial regressive models based on the maximum likelihood estimation include the Akaike information criterion (AIC), the maximized log likelihood (LIK), and the Schwartz criterion (SC). The model with the least AIC, greatest LIK, or least SC has the best goodness-of-fit [25,30].

## 3. Results

### 3.1. Change of Ecological Land

Table 1 shows the changes in ecological land of the Poyang Lake Eco-economic Zone from 1990 to 2005. From Table 1, we can conclude that forestland is the main type of ecological land in the Poyang Lake Eco-economic Zone. Although the percentage of forestland increased from 72.17% in 1990 to 72.58% in 2005, the percentage of wetland decreased from 20.69% in 1990 to 20.42% in 2005. The total area of ecological land in the Poyang Lake Eco-economic Zone decreased by 178.17 km^2^ from 1990 to 2005.

To better reflect the evolutionary characteristics of the spatial disparities of ecological land change, the study time is divided into two sub-stages. At the 95% confidence level, the Moran’s I value of ecological land change from 2000 to 2005 is significantly positive. This result indicates that the high or low values of ecological land changes in the research area are the same as its surrounding counties. From Table 2, we can see that the global Moran’s I value decreased from 0.2091 during the 1990 to 2000 time period to 0.1380 during the 2000 to 2005 period, which implies that the clustering trend of ecological land change in the Poyang Lake Eco-economic Zone is weakening.

### 3.2. Local Spatial Disparities of Ecological Land

#### 3.2.1. Local Moran’s I_i_

To explore in depth the local spatial disparities of ecological land change from 1990 to 2005 in the Poyang Lake Eco-economic Zone, we used the OpenGeoda software package and obtained the results of the local Moran’s I and its significant test during the 1990 to 2000, 2000 to 2005, and 1990 to 2005 time periods (see Table 3).

Table 3 presents the range of local Moran’s I values from 1990 to 2005 of each county in the Poyang Lake Eco-economic Zone (−0.5106, 1.3989). The negative value of the local Moran's I imply that there is spatial heterogeneity in the ecological land. The range of local Moran’s I values of each county in the study area is 1.8825 during the 1990 to 2000 time period. The maximum value of the local Moran’s I is 1.2356 during the 2000 to 2005 time period and 1.3989 during the 1990 to 2005 time period, and all the maximum values are from Nanchang County. The minimum value of the local Moran’s I is −0.8155 during the 1990 to 2000 and is located in Jingdezhen County. The minimum value of the local Moran’s I during the 2000 to 2005 time period is located in Jinxian County. Table 3 also indicates that 12% of the counties during the 1990 to 2000 time period and 19% of the counties during the 2000 to 2005 time period are significantly heterogeneous. The changes in the rate of local Moran’s I values indicate the upward and downward trends, respectively. Table 3 also indicates that the spatial clustering of ecological land change at the county level weakened and the spatial heterogeneity increased.

**Table 1:** Ecological land changes in the Poyang Lake Eco-economic Zone from 1990 to 2005.

Ecological Land Type	1990		2000		2005	
	Area (km^2^)	Percentage (%)	Area (km^2^)	Percentage (%)	Area (km^2^)	Percentage (%)
Forestland	22,044.98	72.17	22,056.81	72.25	22,040.23	72.58
Grassland	2,182.70	7.15	2,165.48	7.09	2,127.52	7.01
Wetland	6,318.69	20.69	6,306.43	20.66	6,200.45	20.42
Ecological land	30,546.37	100.00	30,528.72	100.00	30,368.20	100.00

**Table 2:** Global Moran’s *I* value and its statistical test for ecological land change from 1990 to 2005.

Time period	Moran’s *I*	E(*I*)	Mean	*Z_Score_*
1990~2000	0.2091 *	−0.0323	0.0298	2.2269
2000~2005	0.1380 *	−0.0323	−0.0299	1.9848
1990~2005	0.1646 *	−0.0323	−0.0302	2.0425

Note: * *p* < 0.05.

**Table 3:** Related parameters of the local Moran’s I for ecological land change in the Poyang Lake Eco-economic Zone from 1990 to 2005.

Time period	Minimum	Maximum	Mean	Moran’s *I_i_* (+)	Moran’s *I_i_*(−)	Range
1990~2000	−0.8155	1.0670	0.2025	9.2379	2.7569	1.8825
2000~2005	−0.4629	1.2356	0.1144	5.5991	1.9398	1.6985
1990~2005	−0.5106	1.3989	0.5943	7.4170	2.3153	1.9095

#### 3.2.2. Spatial Association and Distribution Features Based on the Local Moran’s I_i_

**Figure 2:**
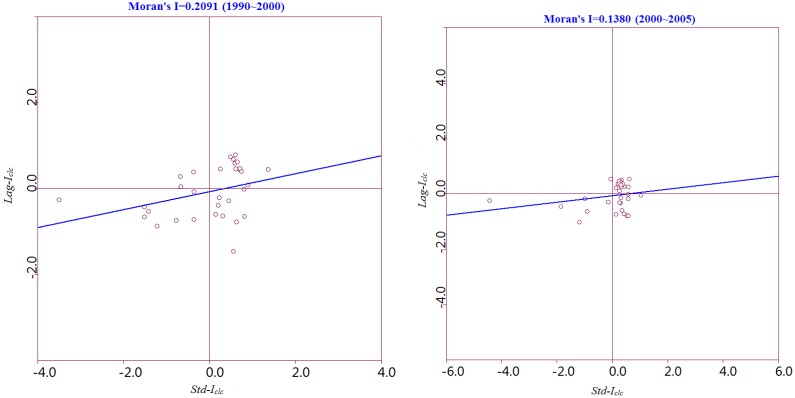
Moran scatter plot of the ecological land at the county level from 1990 to 2005. (Please provide figures with higher resolution.)

If the variable *Z* and spatial variable *W*_z_ at the research unit are calculated and used as the lateral axis and longitudinal axis, we can obtain the Moran scatter plot of ecological land change. Thus, the standardized value (Std-I_clc_) of the research observations is used as the lateral axis, and the spatial lag value (Lag-I_clc_) is the longitudinal axis. The Moran scatter plot of ecological land change composed of the local Moran's *I_i_* for each county in the Poyang Lake Eco-economic Zone is shown in Figure 2.

A positive Std-I_clc_ value indicates that the research unit belongs to those areas that have high values of ecological land changes. A negative Std-I_clc_ belongs to those areas that have low values of ecological land change. The number of areas with a positive Std-I_clc_ value accounts for 72% of the land area during 1990 to 2005 (see Table 4). The positive Lag-I_clc_ value in the Moran scatter plot indicates that the surrounding areas of research unit belong to those areas with high values of ecological land change. From Table 4, we can see that the number of areas with a positive Lag-I_clc_ value account for 40% during 1990 to 2005. The number of areas with a positive Lag-I_clc_ value increased from 44% during 1990 to 2000 to 84% during 2000 to 2005, which implies that number of surrounding areas of research units with a high value of ecological land change increased because of the spatial association. The number of areas with a positive Std-I_clc_ value increased from 31% during 1990 to 2000 to 38% during 2000 to 2005.

**Table 4:** Related parameters and disparity type of the standardized variable *Z* for ecological land change at the county level (%).

Time period	Std-I_clc_ >0	Std-I_clc_ <0	Lag-I_clc_ >0	Lag-I_clc_ <0	H–H		L–L		L–L		H–L	
	Ratio	Ratio	Ratio	Ratio	Comparison ratio	Ratio	Comparison ratio	Ratio	Comparison ratio	Ratio	Comparison ratio	Ratio
1990~2000	68.75	31.25	43.75	56.25	S_+_L_+_	34.38	S_+_L_−_	34.38	S_−_L_−_	21.88	S_−_L_+_	9.38
2000~2005	62.50	37.50	84.38	15.63	S_+_L_+_	59.38	S_+_L_−_	3.13	S_−_L_−_	25.00	S_−_L_+_	12.50
1990~2005	71.88	28.13	40.63	59.38	S_+_L_+_	37.50	S_+_L_−_	34.38	S_−_L_−_	25.00	S_−_L_+_	3.13

Using to the positive or negative attributes of the Std-I_clc_ and Lag-I_clc_ values, we can obtain the four types of areas of ecological land change (Table 4). The four types of areas are referred to as the High-High (H-H) type, which has a positive correlation; the Low-Low (L-L) type, which has a positive correlation; the Low-High (L-H) type, which has a negative correlation; and the High-Low (H-L) type, which has a negative correlation.

From Table 4, we can conclude that the number of areas that belong to the L-L type accounted for 30% during 1990 to 2005 (e.g., the counties of Yugang, Pengze, and Fuliang). This result occurred primarily because of the low urbanization and industrialization in the aforementioned areas. From Table 4, we can see that the number of the areas that belong to the H-H type accounted for 34% during 1990 to 2005, (e.g., Jiujiang, Fuzhoushi, and Xinyushi countie). This result occurred because of the greater urbanization and industrialization level in the aforementioned areas.

**Figure 3:**
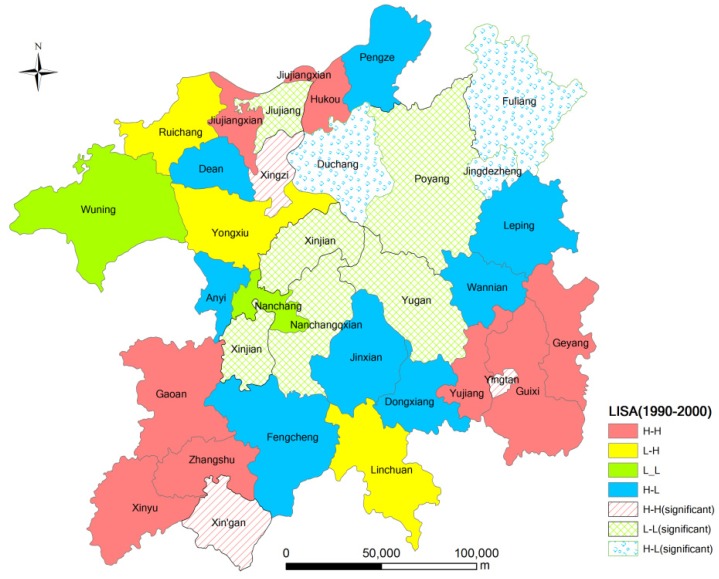
LISA clustering of ecological land change in the Poyang Lake Eco-economic Zone during 1990-2000 at the county level.

**Figure 4:**
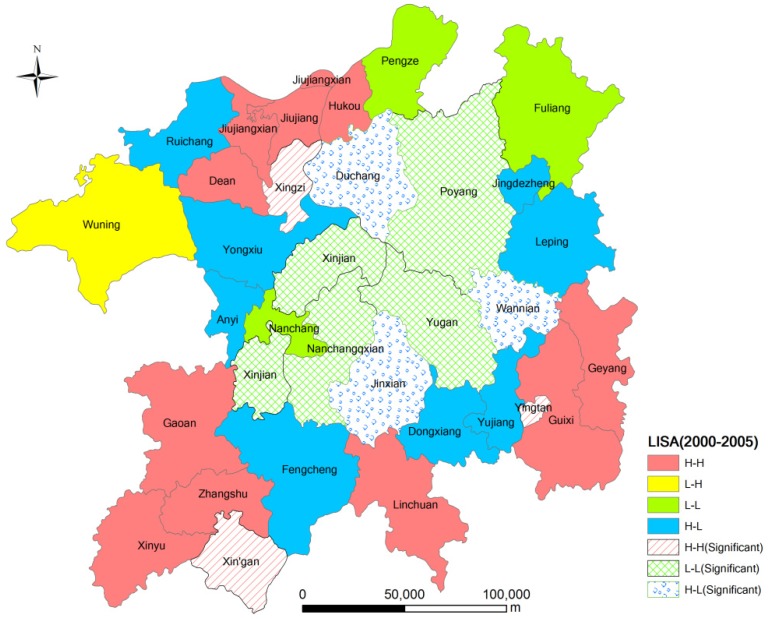
LISA clustering of ecological land change in the Poyang Lake Eco-economic Zone during 2000–2005 at the county level.

To effectively explore the spatial characteristics of ecological land change in the Poyang Lake Eco-economic Zone during 1990 to 2005, LISA clustering maps of two time stages formed by matching the type to which the area belongs with the corresponding spatial location of each county during 1990 to 2000 and during 2000 to 2005 are shown in Figure 3 and Figure 4.

Figure 3 and Figure 4 indicate that the areas that belong to the H-H type have a positive spatial autocorrelation during 1990 to 2000 and during 2000 to 2005, where Std-I_clc_ > 0 and Lag-I_clc_ > 0. The local spatial disparities of ecological land change are stronger with local homogeneity in those areas, where high values are surrounded by neighbors with high values. Table 4 indicates that the proportion of those type of areas increased from 34% during 1990 to 2000 to 59% during 2000 to 2005. Especially at the 5% significance level, the Xingan, Yingtan, and Xinzi counties have a significantly positive relationship for 1990 to 2000 and 2000 to 2005 (Figure 3 and Figure 4).

From Figure 3 and Figure 4, we can conclude that the areas that belong to the L-L type have a positive spatial autocorrelation, where Std-I_clc_ < 0 and Lag-I_clc_ < 0. This result implies that the local spatial disparities of ecological land change is small in those areas, and low values are surrounded by neighbors with low values. The number of L-L-type areas increased from 7 during 1990 to 2000 to 8 during 2000 to 2005. The L-L type area ratio (22%) is less than the average level in the Poyang Lake Eco-economic Zone during 1999 to 2000. All areas that belong to this type are significantly distributed in the surrounding areas of the Poyang Lake, where protection policies of ecological land have been effective in the past 15 years. At the 5% significance level, Poyang and Yugang Counties both have significantly positive relationships during 1990 to 2005 and during 2000 to 2005 (Figure 3 and Figure 4).

The areas that belong to the H-L type exhibit a negative spatial autocorrelation, where Std-I_clc_ > 0 and Lag-I_clc_ < 0. This result implies that clusters of dissimilar values (e.g., locations with high values surrounded by neighbors with low values) forms the hot spots of local heterogeneity. There are three areas that belonged to the H-L type during 1990 to 2000 and four areas that belonged during 2000 to 2005. At the 5% significance level, Duchang and Fuliang Counties have significantly negative relationships during 1990 to 2005 (Figure 3).

The areas that belong to the L-H type have a negative spatial autocorrelation, where Std-I_clc_ < 0 and Lag-I_clc_ > 0 (Figure 3 and Figure 4). This result implies that clusters of dissimilar values (e.g., locations with low values surrounded by neighbors with high values) form cold spots of local heterogeneity. There were 11 L-H types during 1990 to 2000 and 1 during 2000 to 2005.

The above results demonstrate that cluster plot of LISA can measure the spatial heterogeneity and identify the hot and cold spots of spatial clustering in the local space for ecological land change.

### 3.3. Influencing Factors of Ecological Land Change

**Figure 5:**
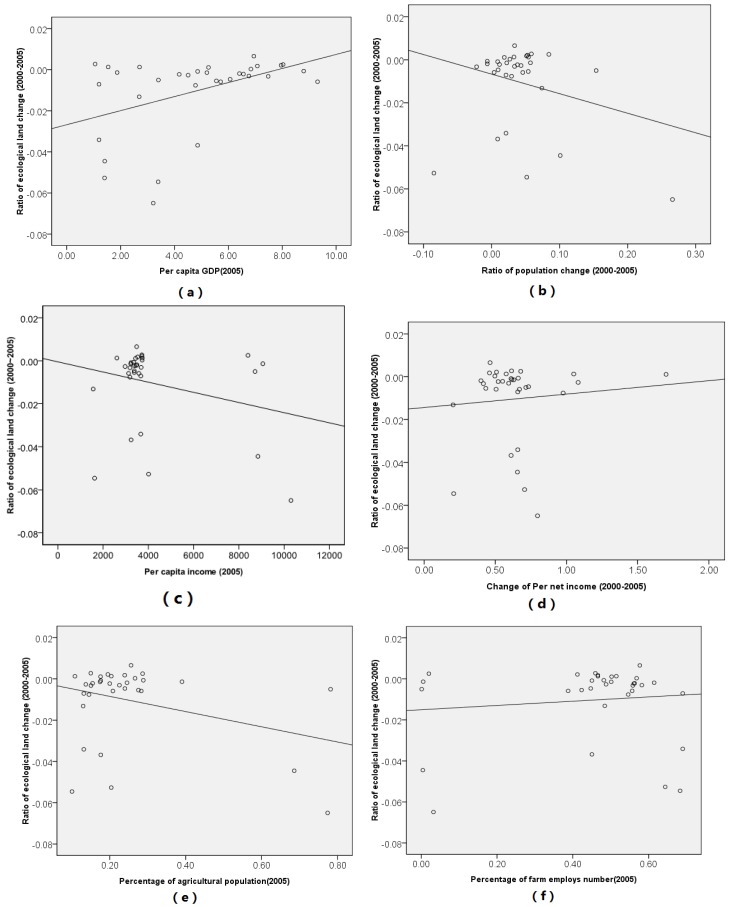
Scatter plot of ecological land change during 2000 to 2005 in Poyang Lake Eco-economic Zone.

To explore the influencing factors of ecological change during 2000 to 2005 in the Poyang Lake Eco-economic Zone, seven independent variables were selected for the standard regression model. Before conducting the regression analysis, we performed a preliminary analysis between independent variables. Preliminary results of the correction analysis between ecological land change and six economic variables can be observed in Figure 5.

From Figure 5, we can conclude that the variables *per capita GDP* and *change of per capita net income* are positively related with ecological land change from 2000 to 2005 in the study area. In contrast, there are positive relationships between the *ratio of population growth*, *percentage of agricultural population*, and ecological land change during 2000 to 2005 in the Poyang Lake Eco-economic Zone (Figure 5).

The coefficient of determination, R^2^, between the independent variables ranges from 0.06 to 0.52, which is less than the critical value of 0.8. All of the independent variables can be incorporated into the three regression models. Table 5 presents the results of statistical tests of three regression models. The SEM has the best goodness-of-fit compared with the other two models because of its lowest AIC, highest LIK, or lowest SC (Table 5). Thus, the SEM is better than the linear regression model and the SLM for analyzing the factors that affect ecological land change in the study area. 

**Table 5:** Statistical tests of three regression models.

Model type	R^2^ or Pseudo R^2^	LIK	AIC	SC
Linear regression model	0.416	93.476	−170.953	−158.981
Spatial lag model	0.459	94.261	−170.522	−157.053
Spatial error model	0.644	101.646	−187.292	−175.319

The results for the parameters of the three regression models are listed in Table 6. The statistical test of linear regression model indicates that there is no significant relationship between the variables and ecological land change in the traditional linear regression model. Compared with the significance of the parameters of three models, we can conclude that the significance level of some parameters increased and two variables, the *percentage of agricultural population* and the *percentage of farm employees number*, are significantly correlated with ecological land change in the SEM (*p* < 0.05). The SEM was used to analyze the driving forces of ecological land in the Poyang Lake Eco-economic Zone.

From the statistical test of spatial error model, we can see that the error parameter, λ, of the SEM passed the significance test, which implies that the ecological land change of the 38 counties in the Poyang Lake Eco-economic Zone is strongly spatially dependent. This result illustrates that the spatially dependent effect of the adjacent areas of ecological land change in the study area exists in the error term and not only contains the interaction of ecological land change between regions but is also present in the space of various complex factors.

From the statistical test of spatial error model, we can conclude that the most influential factor is the variable *per capita GDP*, which implies that ecological land change in Poyang Lake Eco-economic Zone during 2000 to 2005 was primarily induced by economic development, *i.e.*, the higher the economic development level, the greater the changes in ecological land.

Table 6 also demonstrates that the variables *percentage of agricultural population* and *percentage of farm employees number* are the second-most crucial factors that impacted ecological land change in the Poyang Lake Eco-economic Zone during 2000 to 2005. From Table 6, we can see that the variable *mean altitude* is positively related with ecological land change. This result implies that the greater ecological land change is likely to occur in the mountainous area. The spatial lag model demonstrates that the variables *percentage of agricultural population* and *percentage of farm employees number* are negatively related with ecological land change at the 5% significance level. This result implies that the lower the *percentage of agricultural population* and *farm employees number*, the greater the ecological land change. The two variables *percentage of agricultural population* and *percentage of farm employees number* are also expressions of the level of urbanization. Urbanization makes more farmers engage in non-agricultural industries and move out of rural areas. In those more-urbanized area, more ecological land disappeared because of urban construction. The result also proves that the spatial autoregressive model can find some potentially influencing drivers of ecological land change compared with the traditional linear regression model.

**Table 6:** Parameters of the three regression models for ecological land change in the Poyang Lake Eco-economic Zone during 2000 to 2005.

**Variable**	**Coefficient**	**Std. Error**	**t Statistic**	**Probability**
(A) Linear regression model				
Constant	0.016	0.029	0.540	0.594
*Per capita* GDP (10^4^￥/per)	0.002	0.001	1.881	0.072
Ratio of population growth (2000–2005) (%)	−0.037	0.079	−0.474	0.639
Mean altitude (m)	6.42E−05	4.44E−05	1.447	0.160
Percentage of agricultural population (%)	−0.053	0.037	−1.435	0.164
Percentage of farm employees number (%)	−0.055	0.0339	−1.657	0.110
*Per capita* net income (￥/per)	−2.41E−06	3.66E−06	−0.659	0.516
Change of *per* *capita* net income (2000–2005) (￥/per)	0.008	0.011	0.725	0.475
**Variable**	**Coefficient**	**Std. Error**	**Z Statistic**	**Probability**
(B) Spatial lag model				
Ρ	−0.366	0.239	−1.529	0.126
Constant	0.019	0.025	0.793	0.428
*Per capita* GDP (10^4^￥/per)	0.003	0.001	2.725	0.006
Ratio of population growth (2000–2005) (%)	−0.055	0.067	−0.823	0.411
Mean altitude (m)	7.70E−05	3.86E−05	1.995	0.046
Percentage of agricultural population (%)	−0.056	0.0312	−1.779	0.075
Percentage of farm employees number (%)	−0.072	0.029	−2.495	0.013
*Per capita* net income (￥/per)	−3.15E−06	3.15E−06	−0.999	0.317
Change of *per* *capita* net income (2000–2005) (￥/per)	0.009	0.009	0.963	0.335
**Variable**	**Coefficient**	**Std. Error**	**Z Statistic**	**Probability**
(C) Spatial error model				
Λ	−0.828	0.240	−3.447	0.001
Constant	0.019	0.020	0.937	0.348
*Per capita* GDP (10^4^￥/per)	0.004	0.000	3.888	0.000
Ratio of population growth (2000–2005) (%)	−0.061	0.062	−0.986	0.324
Mean altitude(m)	4.37E−05	3.20E−05	1.3666	0.172
Percentage of agricultural population (%)	−0.069	0.029	−2.362	0.018
Percentage of farm employees number (%)	−0.070	0.022	−3.132	0.002
*Per capita* net income (￥/per)	−8.63E−07	2.44E−06	−0.354	0.724
Change of *per* *capita* net income (2000–2005) (￥/per)	0.007	0.006	1.170	0.242

## 4. Discussion and Conclusions

The ESDA, which measures spatial association, can solve the spatial relationship problem. It provides stronger support for the quantitative analysis of spatial disparities in ecological land change. We can conclude that the ESDA is an effective method for measuring spatial patterns of ecological land changes and can be used to explore the distribution characteristics, local heterogeneity, and local homogeneity by comparing it with the general clustering analysis.

The regional distribution of ecological land change had significant clustering characteristics during 1990 to 2005 in the Poyang Lake Eco-economic Zone, which indicates that the ecological land changes of the research unit and its surrounding areas are faster than in other areas. Moran’s I value of ecological land change had a declining trend during 1990 to 2005, which implies that the clustering trend of ecological land changes weakened in the study area.

Based on the compound attribute of the Std-I_clc_ and Lag-I_clc_ values, four disparity types and two spatial associations were identified. Those types and their surrounding areas with less ecological land change are significantly distributed around the Poyang Lake.

The spatial dependence of ecological land change may be related to the spillover effects of the regional economy, regional population flow and concentration, the diffusion effect of urbanization of ecological land, and other factors [29]. The results of the spatial autoregressive model demonstrate that the ecological land change in the Poyang Lake Eco-economic Zone is primarily induced by the *per capita* GDP, percentage of agricultural population, and percentage of farm employees’ number. The high urbanization rate and economic development level may be the main causes for ecological land change. Meanwhile, those results also demonstrate that the spatial autoregressive model can better distinguish the main drivers of ecological land change in the study area.

In this paper, the influential factors of ecological land changes in China were analyzed using the classic linear regression model, and the effects of spatial autocorrelation were ignored. The spatial regression model provides a statistically reasonable solution. Compared with the classic linear regression model, there is no spatial autocorrelation of the residuals in the SEM, and it has a better goodness-of-fit. The SEM can reveal various factors of ecological land change and effectively solve the problem of ecological land change that is related to the spatial distribution. The estimated results of are spatial autoregressive model more accurate and reliable. This paper also demonstrated that some potential driving forces for economical land change can be found by applying the spatial autoregressive model rather than the general linear regressive analysis.

Although economic factors are always the most important driving force of land use changes, the mechanisms of land use changes cannot be comprehensively and systematically reflected from only an economic angle [5,11]. There are many other factors that influence ecological land change, such as land and agricultural policy in China, which includes the conversion of cropland to forest and grassland project, the agricultural subsidy policy, and the cultivated land protection policy. Therefore, considering how to determine policies is very important for exploring the mechanisms of ecological land in the spatial autoregressive model.
